# The clinical characteristics and prognosis in chronic obstructive pulmonary disease patients with anorexia

**DOI:** 10.1080/07853890.2025.2590186

**Published:** 2025-11-21

**Authors:** Qing Song, Dan Peng, Ping Zhang, Tao Li, Cong Liu, Ling Lin, Yuqin Zeng, Ping Chen

**Affiliations:** ^a^ Department of Pulmonary and Critical Care Medicine, the Second Xiangya Hospital, Central South University, Changsha, Hunan, China; ^b^ Research Unit of Respiratory Disease, Central South University, Changsha, Hunan, China; ^c^ Clinical Medical Research Center for Pulmonary and Critical Care Medicine, Hunan Province, Changsha, Hunan, China; ^d^ Diagnosis and Treatment Center of Respiratory Disease, Central South University, Changsha, Hunan, China; ^e^ The Fourth People’s Hospital of Nanchang County, Nanchang, Jiangxi, China

**Keywords:** Chronic obstructive pulmonary disease, anorexia, exacerbation, symptom scores, pulmonary function

## Abstract

**Background:**

Anorexia is a common problem among patients with chronic obstructive pulmonary disease (COPD). This study aimed to analyze the clinical characteristics and prognosis of COPD patients with anorexia.

**Methods:**

This prospective cohort study included patients registered in the RealDTC study between May 2023 and February 2024. Demographic on COPD Assessment Test (CAT) and modified Medical Research Council (mMRC) scores, pulmonary function, number of exacerbations, inhalation therapy, and the section of anorexia/cachexia subscale (A/CS)-12 of Functional Assessment of Anorexia/Cachexia Therapy (FAACT) scores were collected. A FAACT A/CS-12 score of ≤ 30 was used to identify patients with anorexia. All patients were followed up for one year to collect exacerbations, and mortality.

**Results:**

A total of 758 patients with COPD were enrolled, 132 (17.4%) of whom had anorexia. Patients with anorexia had higher age, CAT and mMRC scores, number of exacerbations, and worse pulmonary function. Logistic regression analysis showed that CAT scores of 10–19 (OR = 2.867, 95% CI = 1.423–5.773), 20–29 (OR = 6.932, 95% CI = 3.234–14.857) and ≥ 30 (OR = 67.355, 95% CI = 7.221–628.271), and number of hospitalizations ≥ 1 (OR = 2.041, 95% CI = 1.347–3.093) were independent risk factors for anorexia (*p* < 0.05). In addition, patients with anorexia experienced more future exacerbations, frequent exacerbations, and hospitalizations (*p* < 0.05). The ROC curves showed that FAACT A/CS-12 scores had a predictive capacity for future exacerbation, frequent exacerbation, and hospitalization.

**Conclusions:**

The COPD patients with anorexia had worse pulmonary function, higher symptoms burden, and risk of exacerbation and hospitalization.

## Introduction

Chronic obstructive pulmonary disease (COPD) is a common chronic respiratory disease which characterized by persistent respiratory symptoms and airflow limitation. COPD is associated with high morbidity and mortality and has become the third leading cause of death [1,2]. The prevalence of COPD in individuals over 40 years of age is 13.7%, and that in individuals over 60 years of age is more than 27% in the Chinese population [3]. Therefore, it is crucial to prevent and treat the disease.

Anorexia defined as a reduced in desire to eat and is a common issue in many diseases such as cancer, chronic renal failure, and liver failure. It is strongly associated with poor health-related quality of life, higher risk of hospitalization, and mortality [4,5]. In addition, anorexia is a common problem in patients with severe and very severe COPD and holds prognostic significance. In fact, anorexia or lack of appetite can lead to weight loss, muscle wasting, and even cachexia in patients with COPD, which can aggravate the progression of COPD and contribute to a poor prognosis [6]. Grönberg et al. [7] examined the dietary problems in patients with severe COPD and found that the most frequently reported issue was anorexia, which was associated with smoking habits and sex. There is little doubt that anorexia, as a symptom closely associated with poor quality of life and prognosis in patients with COPD, is often overlooked in clinical practice. Moreover, research examining the relationship between anorexia and symptom scores, pulmonary function, risk of exacerbation, and hospitalization in patients with COPD remains limited. However, it is crucial to investigate the clinical characteristics and prognosis of COPD patients with anorexia for the better prevention and treatment of the disease.

Therefore, in this study, we first analyzed the clinical characteristics of COPD patients with anorexia, including demographics data, symptom scores, pulmonary function, risk of exacerbation and hospitalization. Furthermore, we explored the independent factors associated with anorexia in COPD patients. Finally, we conducted a one-year follow-up to explore the prognosis of COPD patients with anorexia, including number of exacerbations, frequent exacerbations, hospitalizations, and mortality.

## Patients and methods

### Study participants

This prospective study involved patients enrolled in the RealDTC study [8] between May 2023 and February 2024. Patients were diagnosed with COPD according to the Global Initiative for Chronic Obstructive Lung Disease (GOLD) 2023 report: a post-bronchodilator ratio of forced expiratory volume in 1 s to forced vital capacity (FEV1/FVC) of < 0.70 [6]. We excluded patients with lung cancer; active tuberculosis; bronchiectasis; pneumonia; pulmonary fibrosis; and severe heart (New York Heart Association class III or IV heart failure, left ventricular ejection fraction < 35%, unstable angina or acute myocardial infarction within the past 6 months, or severe arrhythmias requiring intervention.), liver (Cirrhosis with a Child-Pugh score of B or C, a history of portal hypertension, hepatic encephalopathy, or serum albumin level < 30 g/L), or kidney disease (Chronic kidney disease stage 4 or 5, estimated glomerular filtration rate < 30 mL/min/1.73m^2^ or the need for renal replacement therapy) [9–11]. Patients who were unable to complete the questionnaire were also excluded.

This study was approved by an institutional review board from the Second Xiangya Hospital of Central South University and conducted following the Declaration of Helsinki (Date: 29 December 2016; Number: 2016076). All patients have given written informed consent to participate in the research.

### Data collection

Baseline data were collected on sex, age, body mass index (BMI), education level, smoke status, smoking (pack/year), biofuel exposure, COPD Assessment Test (CAT) score, modified Medical Research Council (mMRC) score, Clinical COPD Questionnaire (CCQ) score, FEV1, FEV1%pred and FVC, GOLD grades, GOLD groups, number of exacerbations, and hospitalizations in the past year, and inhalation therapy regimens. The section of the anorexia/cachexia subscale (A/CS)-12 of the Functional Assessment of Anorexia/Cachexia Therapy (FAACT) scores were collected at the patient’s initial hospital visit.

All patients were followed up for one year to collect the number of exacerbations, frequent exacerbations, hospitalizations, and all-cause mortality.

### Anorexia scores

After registering for academic research purposes and obtaining a license agreement, we accessed the questionnaire from the web of FACIT.org website (http://www.facit. org/FACITOrg/Questionnaires), and downloaded the FAACT questionnaire and Chinese version (version 4) (Supplement Figure 1). The FAACT A/CS-12 consists of 12 questions related to appetite and food intake, allowing both qualitative and quantitative assessment of anorexia. Each question is rated on a 5-point Likert scale (not at all = 0, a little bit = 1, somewhat = 2, quite a bit = 3, very much = 4), with total scores ranging from 0 to 48. Lower scores indicate poor appetite. The FAACT A/CS-12 questionnaire was completed based on patients’ experiences with appetite over the previous 7 days. An FAACT A/CS-12 score of ≤30 has been used as a diagnostic threshold for anorexia [12–14].

**Figure 1.**
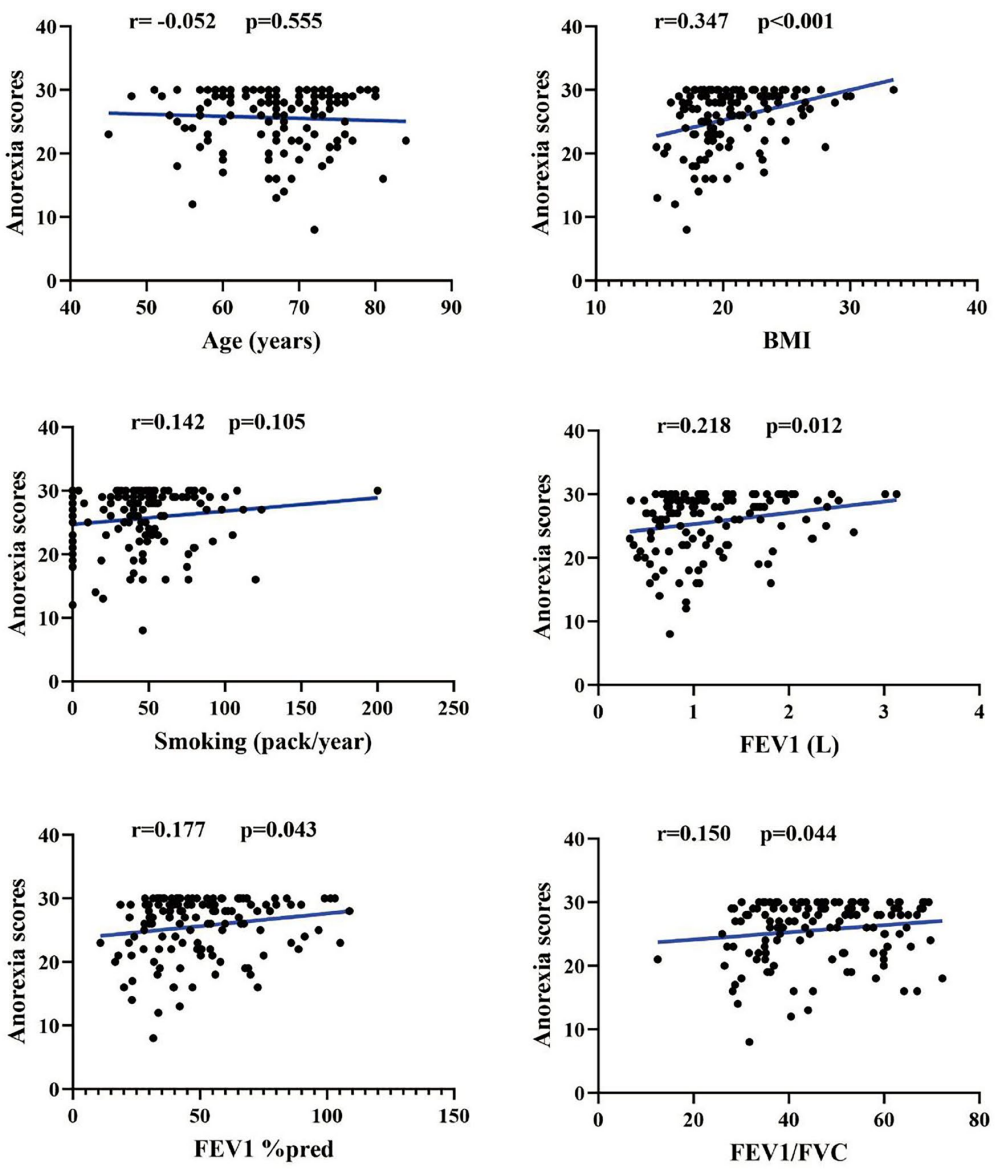
The correlation between anorexia scores and age, BMI, smoking, FEV1, FEV1%pred, and FEV1/FVC in COPD patients with anorexia. BMI: Body Mass Index; COPD: Chronic Obstructive Pulmonary Disease; FEV1: Forced Expiratory Volume in the first second; FEV1%pred: Forced Expiratory Volume in the first second predicted of percentage; FVC: Forced Vital Capacity.

### Variable definition

An exacerbation is COPD progression that requires antibiotics, or oral corticosteroids, or hospitalization [15]. Frequent exacerbations were defined as ≥2 exacerbations per year [16]. A current-smoker has had smoking exposure of ≥ 10 pack/years, and a former-smoker has had ≥ 10 pack/years but has not smoked for more than 6 months [17]. Biofuel exposure was defined as the use of biomass fuels (wood, grass, charcoal, or crop residues) for cooking or heating for at least 2 h per day for at least one year [18]. The patients were assigned into ABE groups according to GOLD 2023 report [6]: group A, 0 to 1 exacerbation per year, no hospitalization, CAT scores of <10, and mMRC scores of 0 to 1; group B, 0 to 1 exacerbation per year, no hospitalization, CAT scores of ≥10, or mMRC scores of ≥2; and group E, exacerbations ≥2, or hospitalization ≥1 per year. GOLD grades were based on the post-bronchodilator FEV1%pred according to GOLD 2023 report [6]: GOLD 1, FEV1 ≥ 80% pred; GOLD 2, FEV1 50–79% pred; GOLD 3, FEV1 30–49% pred; GOLD 4, FEV1 < 30% pred. Prescription outcomes, including adjustment treatment was defined as changes in the inhalation therapy drugs or stopped inhalation therapy drugs for more than three months during one year of follow-up [19]. Given that some patients adjusted inhalation therapy regimens during the follow-up, we incorporated this variable (prescription outcomes) as a confounding factor in the multivariable logistic regression model for prognostic analysis.

### Sample size calculated

We used PASS software (version 15.0.5) to calculate the required sample size. In the first 3 months, 400 patients with COPD were enrolled, of whom 61 had FAACT A/CS-12 scores of ≤ 30, with an estimated prevalence of 15.25% in the patients with anorexia. A confidence level of 0.95, and a two-sided confidence interval width of 0.06. Accounting for a 20% dropout rate, the minimum required sample size was calculated to be 730.

### Statistical analysis

Statistical analysis was performed using SPSS 26.0 (IBM, Armonk, USA) and Free Statistics software version 1.7.1 (Beijing, China). Continuous variables including FAACT A/CS-12 scores, age, BMI, FEV1, FEV1%pred, FEV1/FVC, CAT and CCQ scores were normal distribution and homogeneous variance, and expressed as mean ± standard deviation, and analyzed using the *t*-test. Other continuous variables, including smoking (pack/year), mMRC scores, number of exacerbations and hospitalizations were presented as median and interquartile range and analyzed using non-parametric tests. Categorical variables including sex, education level. smoke status, biofuel exposure, GOLD grades, GOLD groups, inhalation therapy, exacerbation, frequent exacerbation, hospitalization, mortality, and prescription outcome were analyzed using the chi-square test. Logistic regression was used to analyzed the independently relative factors for COPD patients with anorexia and the variables of age, BMI, education level, CAT scores, exacerbation in the past year, hospitalization in the past year, mMRC scores, and GOLD grades were included in the model and odds ratios (OR) and 95% confidence intervals (95% CI) were calculated. In order to analyze the prognosis, the confounding factors including education level, therapy, prescription outcome, age, sex, BMI, smoke status, biofuel exposure, FEV1%, FEV1/FVC, CAT, mMRC, and exacerbations in the past year were included in the logistic regression model. Pearson correlation analysis was used to assess the relationship between anorexia scores and clinical characteristics of patients with COPD, and correlation coefficients (r) were reported. Receiver operating characteristic (ROC) curves were generated, and areas under the curve (AUC) were compared using the DeLong’s test. The cut off value was determined at the point where the Youden index (sensitivity + specificity − 1) is maximized. A *p*-value of <0.05 was considered statistically significant.

## Results

### The clinical characteristics of the COPD patients with anorexia

In total, 768 patients with COPD were included in the study. After 10 patients were excluded because of incomplete data, 758 patients were included in the final analysis (Supplementary Figure 2). The mean anorexia score was 38.2 ± 7.0, and 132 patients (17.4%) were identified as having anorexia. The mean age of the participants was 64.9 ± 8.0 years, and 91.8% were male. The mean CAT score was 13.8 ± 6.2, and the mean FEV_1_%pred was 56.0 ± 1.4 (Table 1).

**Table 1.** The baseline clinical characteristics of the total patients with COPD.

Variables	Total (*n* = 758)
FAACT A/CS-12 scores, (Mean ± SD)	38.2 ± 7.0
Anorexia, *n* (%)	
Yes	132 (17.4)
No	626 (82.6)
Age, (years)	64.9 ± 8.0
Sex, *n* (%)	
Male	696 (91.8)
Female	62 (8.2)
Education level, *n* (%)	
Elementary school	279 (36.8)
Junior high school	313 (41.3)
High school	127 (16.8)
University	39 (5.1)
BMI, (kg/m^2^), (Mean ± SD)	22.5 ± 3.6
Smoke status, *n* (%)	
Never-smoker	132 (17.4)
Former-smoker	268 (35.4)
Current-smoker	358 (47.2)
Smoking, (pack/year) (Median, IQR)	42.0 (25.0, 57.8)
Biofuel exposure, *n* (%)	
Yes	164 (21.6)
No	594 (78.4)
FEV1, (Mean ± SD)	1.4 ± 0.6
FEV1 %pred, (Mean ± SD)	56.0 ± 1.4
FEV1/FVC, (Mean ± SD)	50.0 ± 2.8
GOLD grades, *n* (%)	
1	109 (14.4)
2	320 (42.3)
3	246 (32.5)
4	83 (10.8)
GOLD groups, *n* (%)	
A	145 (19.1)
B	247 (32.6)
E	366 (48.3)
CAT, (Mean ± SD)	13.8 ± 6.2
CAT, *n* (%)	
<10	201 (26.6)
10–19	426 (56.3)
20–29	123 (16.2)
≥30	7 (0.9)
mMRC, (Median, IQR)	2.0 (1.0, 2.0)
mMRC, *n* (%)	
01	306 (40.4)
≥2	451 (59.6)
CCQ, (Mean ± SD)	20.7 ± 7.3
Therapy, *n* (%)	
LAMA	49 (6.5)
LABA+ICS	39 (5.1)
LABA+LAMA	213 (28.1)
LABA+LAMA+ICS	437 (57.7)
^♥^ Others	20 (2.6)
Exacerbations in the past year, (Median, IQR)	1.0 (0.0, 3.0)
Exacerbations in the past year, *n* (%)	
0	311 (41.0)
1	166 (21.9)
≥ 2	281 (37.1)
Hospitalizations in the past year (Median, IQR)	0.0 (0.0, 1.0)
Hospitalizations in the past year, *n* (%)	
0	480 (63.3)
≥1	278 (36.7)

Notes. ^♥^ Others included SAMA, SABA, SAMA+SABA, LAMA+ICS, ICS, and no inhalation therapy.

Abbreviations: BMI: Body Mass Index; COPD: Chronic Obstructive Pulmonary Disease; CAT: COPD Assessment Test; FEV1: Forced Expiratory Volume in one second; FEV1%pred: Forced Expiratory Volume in the first second predicted of percentage; FVC: forced vital capacity; GOLD: Global Initiative for Chronic Obstructive Lung Disease; ICS: inhaled corticosteroids; IQR: interquartile range; LAMA: long-acting muscarinic antagonist; LABA: long-acting β2-agonist; mMRC: modified Medical Research Council; SD: Standard Deviation; CCQ: Clinical COPD Questionnaire; A/CS: anorexia/cachexia subscale; FAACT: Functional Assessment of Anorexia/Cachexia Therapy; SABA: short acting beta agonist; SAMA: short-acting muscarinic antagonists.

The COPD patients with anorexia had a higher age, CAT scores, mMRC scores, and CCQ scores, number of exacerbations, and hospitalizations in the past year, and lower BMI, FEV1, FEV1%pred, and FEV1/FVC. In addition, CAT scores of 20–29 and ≥ 30, mMRC scores ≥ 2, GOLD grades 3–4, GOLD group E, and elementary school education level were higher in patients with anorexia (*p* < 0.05). Furthermore, the proportion of number of exacerbations ≥ 2 and hospitalization ≥ 1 was higher in patients with anorexia (*p* < 0.05) (Table 2).

**Table 2.** The clinical characteristics of the COPD patients with anorexia.

Variables	Non-anorexia (*n* = 626)	Anorexia (*n* = 132)	*p* value
FAACT A/CS-12 scores, (Mean ± SD)	40.9 ± 3.6	25.6 ± 4.7	**<0.001**
Age, (years) (Mean ± SD)	64.6 ± 8.0	66.6 ± 7.5	**0.007**
Sex, n (%)			0.263
Male	578 (92.3)	118 (89.4)	
Female	48 (7.7)	14 (10.6)	
Education level, n (%)			**0.009**
Elementary school	216 (34.5)	63 (47.7)	
Junior high school	274 (43.8)	39 (29.5)	
High school	102 (16.3)	25 (18.9)	
University	34 (5.4)	5 (3.8)	
BMI, (kg/m^2^) (Mean ± SD)	22.9 ± 3.5	20.8 ± 3.5	**<0.001**
Smoke status, n (%)			0.939
Never-smoker	109 (17.4)	23 (17.4)	
Former-smoker	223 (35.6)	45 (34.1)	
Current-smoker	294 (47)	64 (48.5)	
Smoking, (pack/year) (Median, IQR)	41.0 (25.0, 57.0)	44.5 (28.0, 59.2)	0.260
Biofuel exposure, n (%)			0.570
Yes	133 (21.2)	31 (23.5)	
No	493 (78.8)	101 (76.5)	
FEV1, (Mean ± SD)	1.4 ± 0.6	1.2 ± 0.6	**<0.001**
FEV1 %pred, (Mean ± SD)	57.3 ± 21.2	49.5 ± 21.1	**<0.001**
FEV1/FVC, (Mean ± SD)	50.7 ± 12.7	46.5 ± 12.6	**<0.001**
GOLD grades, n (%)			**0.002**
1	96 (15.3)	13 (9.8)	
2	278 (44.4)	42 (31.8)	
3	190 (30.4)	56 (42.4)	
4	62 (9.9)	21 (15.9)	
GOLD groups, n (%)			**<0.001**
A	139 (22.2)	6 (4.5)	
B	212 (33.9)	35 (26)	
E	275 (43.9)	91 (69.5)	
CAT, (Mean ± SD)	12.8 ± 5.6	18.4 ± 6.7	**<0.001**
CAT, n (%)			**<0.001**
< 10	191 (30.6)	10 (7.6)	
10–19	356 (57)	70 (53)	
20–29	77 (12.3)	46 (34.8)	
≥ 30	1 (0.2)	6 (4.5)	
mMRC, (Median, IQR)	2.0 (1.0, 2.0)	2.0 (2.0, 3.0)	**<0.001**
mMRC, n (%)			**<0.001**
0-1	280 (44.8)	26 (19.7)	
≥ 2	345 (55.2)	106 (80.3)	
CCQ, (Mean ± SD)	19.7 ± 6.8	25.6 ± 7.7	**<0.001**
Therapy, n (%)			0.223
LAMA	44 (7)	5 (3.8)	
LABA+ICS	32 (5.1)	7 (5.3)	
LABA+LAMA	180 (28.8)	33 (25)	
LABA+LAMA+ICS	351 (56.1)	86 (65.2)	
^♥^ Others	19 (3)	1 (0.8)	
Exacerbations in the past year, (Median, IQR)	1.0 (0.0, 2.0)	2.0 (1.0, 5.0)	**<0.001**
Exacerbations in the past year, n (%)			**<0.001**
0	281 (44.9)	30 (22.7)	
1	138 (22)	28 (21.2)	
≥ 2	207 (33.1)	74 (56.1)	
Hospitalizations in the past year (Median, IQR)	0.0 (0.0, 1.0)	1.0 (0.0, 2.0)	**<0.001**
Hospitalizations in the past year, n (%)			**<0.001**
0	425 (67.9)	55 (41.7)	
≥ 1	201 (32.1)	77 (58.3)	

Notes: ^♥^ Others included SAMA, SABA, SAMA+SABA, LAMA+ICS, ICS and no inhalation therapy. The bold *p*-value indicates statistical significance.

Abbreviations: BMI: Body Mass Index; COPD: Chronic Obstructive Pulmonary Disease; CAT: COPD Assessment Test; FEV1: Forced Expiratory Volume in one second; FEV1%pred: Forced Expiratory Volume in the first second predicted of percentage; FVC: forced vital capacity; GOLD: Global Initiative for Chronic Obstructive Lung Disease; ICS: inhaled corticosteroids; IQR: interquartile range; LAMA: long-acting muscarinic antagonist; LABA: long-acting β2-agonist; mMRC: modified Medical Research Council; SD: Standard Deviation; CCQ: Clinical COPD Questionnaire; A/CS: anorexia/cachexia subscale; FAACT: Functional Assessment of Anorexia/Cachexia Therapy; SABA: short acting beta agonist; SAMA: short-acting muscarinic antagonists.

### Factors correlated with anorexia among COPD patients

Logistic regression analysis showed that CAT scores of 10–19 (OR = 2.867, 95%CI = 1.423–5.773), 20–29 (OR = 6.932, 95%CI = 3.234–14.857), and ≥30 (OR = 67.355, 95%CI = 7.221–628.271), and number of hospitalizations ≥ 1 (OR = 2.041, 95%CI = 1.347–3.093) were independent risk factors for anorexia among COPD patients. BMI was negatively associated with anorexia, with an OR = 0.873, 95%CI = 0.821–0.929 (*p* < 0.05) (Table 3).

**Table 3.** Multivariate analysis of independent relative factors for COPD patients with anorexia.

Variables	OR	95% CI	*p*-value
BMI	0.873	0.821–0.929	**<0.001**
CAT			
<10	Reference		
10–19	2.867	1.423–5.773	**0.003**
20–29	6.932	3.234–14.857	**<0.001**
≥30	67.355	7.221–628.271	**<0.001**
Hospitalizations in the past year			
0	Reference		
≥ 1	2.041	1.347–3.093	**0.001**

Notes: Variables in the logistic regression model: age, BMI, education level, CAT scores, exacerbation in the past year, hospitalization in the past year, mMRC scores, and GOLD grades. The bold *p*-value indicates statistical significance.

Abbreviations: BMI: Body Mass Index; COPD: Chronic Obstructive Pulmonary Disease; CAT: COPD Assessment Test; GOLD: Global Initiative for Chronic Obstructive Lung Disease; mMRC: modified Medical Research Council; OR: Odds Ratio; 95% CI: 95% Confidence Interval.

### Correlation between anorexia scores and clinical characteristics in COPD patients with anorexia

**Figure 2.**
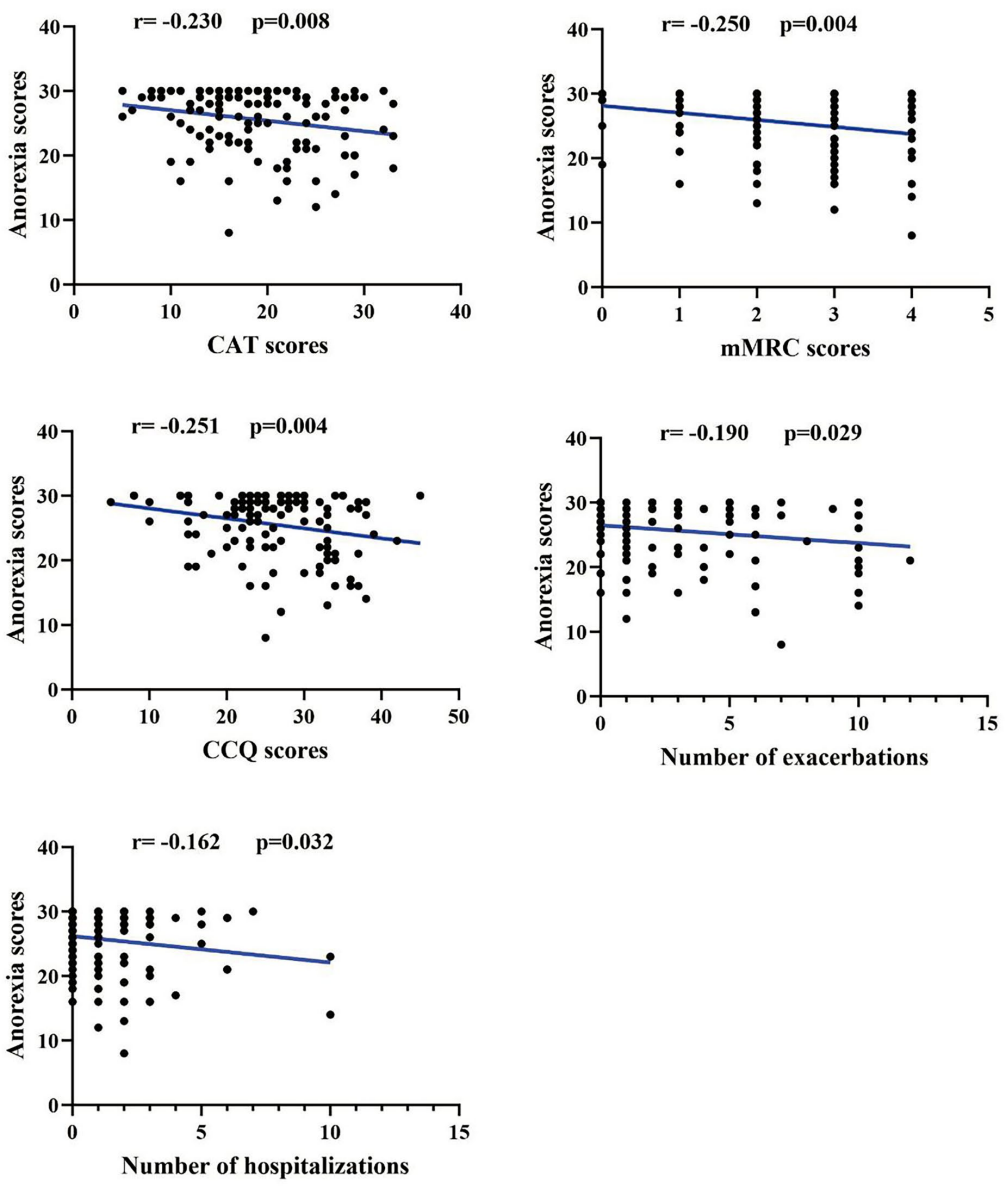
Correlation between anorexia scores and CAT, mMRC, and CCQ scores, number of exacerbations, and hospitalizations in the past year. COPD: Chronic Obstructive Pulmonary Disease; CCQ: Clinical COPD Questionnaire; CAT: COPD Assessment Test; mMRC: modified Medical Research Council.

Correlation analysis showed that BMI, FEV1, FEV1%pred, and FEV1/FVC were positively associated with anorexia scores, with correlation coefficients of 0.347, 0.218, 0.177, and 0.150, respectively (*p* < 0.05) (Figure 1). By contrast, CAT scores, mMRC scores, CCQ scores, number of exacerbations, and hospitalizations in the past year were negatively associated with anorexia scores, with correlation coefficients of −0.230, −0.250, −0.251, −0.190, and −0.162, respectively (*p* < 0.05) (Figure 2).

### The prognosis of COPD patients with anorexia

After one year of follow-up, prognostic data were available for 728 patients, among whom 125 (17.2%) had anorexia. After control the confounding factors, including education level, inhalation therapy, prescription outcome, age, sex, BMI, smoke status, biofuel exposure, FEV1%pred, FEV1/FVC, CAT, mMRC, and exacerbations in the past year. Patients with anorexia experienced higher future exacerbations, frequent exacerbations, and hospitalizations than patients without anorexia (*p* < 0.05) (Table 4 and Supplement Tables 1–3).

**Table 4.** The future exacerbation and mortality of COPD patients with anorexia during one year of follow-up.

Variables	Total (*N* = 728)	Non-anorexia (*n* = 603)	Anorexia (*n* = 125)	*p*-value
Exacerbations during one year, (Median, IQR)	0.0 (0.0, 1.0)	0.0 (0.0, 1.0)	1.0 (0.0, 2.0)	**<0.001**
Exacerbations, n (%)				**<0.001**
No	440 (61.0)	388 (64.8)	52 (42.6)	
Yes	281 (39.0)	211 (35.2)	70 (57.4)	
Frequent exacerbations, n (%)				**<0.001**
No	552 (76.6)	480 (80.1)	72 (59.0)	
Yes	169 (23.4)	119 (19.9)	50 (41.0)	
Hospitalizations during one year, (Median, IQR)	0.0 (0.0, 1.0)	0.0 (0.0, 0.0)	0.0 (0.0, 1.0)	**<0.001**
Hospitalizations, n (%)				**<0.001**
No	533 (73.9)	468 (78.1)	65 (53.3)	
Yes	188 (26.1)	131 (21.9)	57 (46.7)	
Mortality, n (%)	7 (1.0)	4 (0.7)	3 (2.4)	0.102
prescription outcome, n (%)				**0.018**
Adjustment treatment	138 (19.1)	124 (20.7)	14 (11.5)	
Continuous using	583 (80.9)	475 (79.3)	108 (88.5)	

The bold *p*-value indicates statistical significance.

Abbreviations: COPD: Chronic Obstructive Pulmonary Disease; IQR: interquartile range.

**Figure 3.**
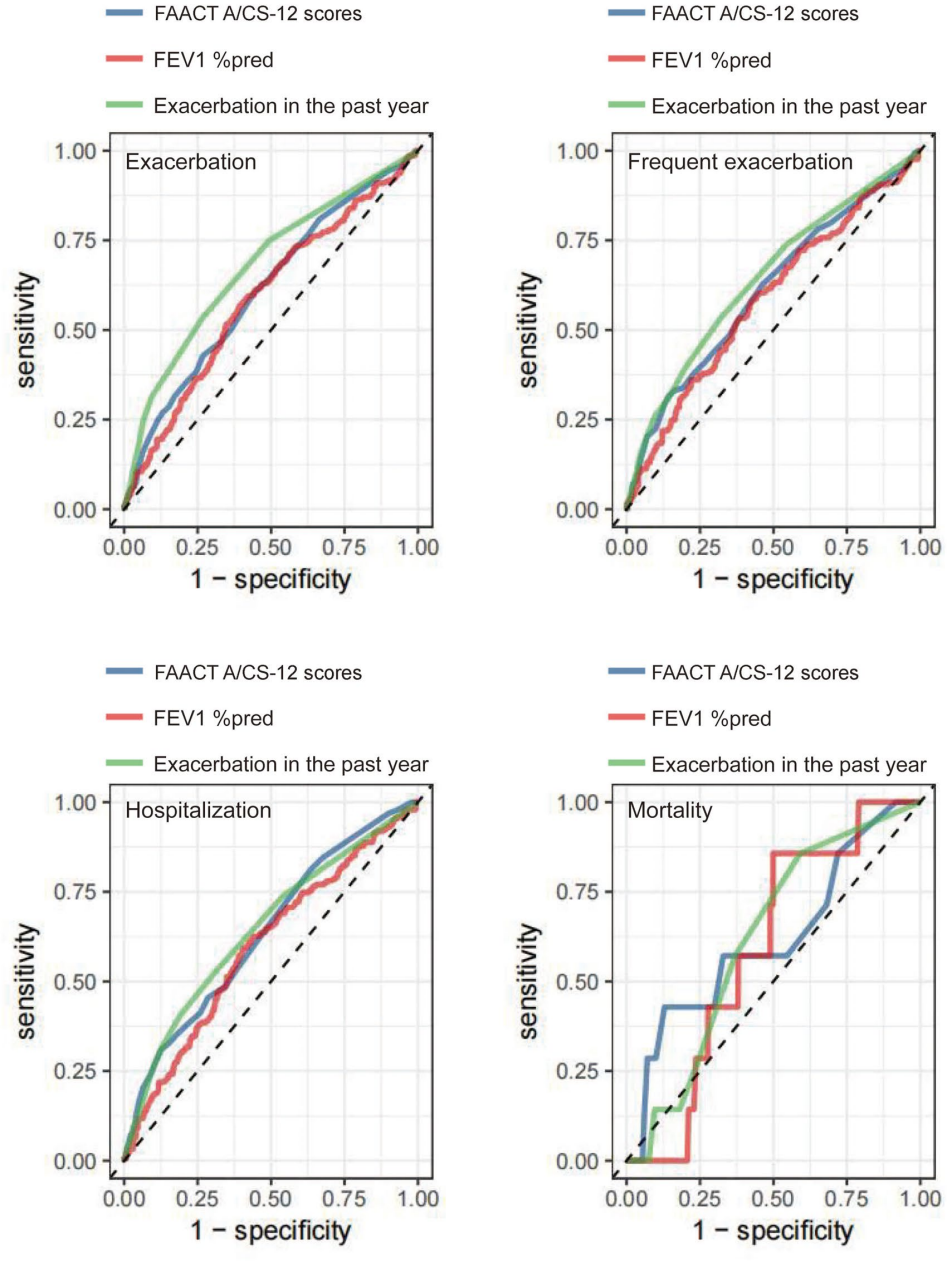
ROC curves of the different indicators predicted exacerbation and mortality in COPD patients. COPD: Chronic Obstructive Pulmonary Disease; FEV1%pred: Forced Expiratory Volume in the first second predicted of percentage; ROC: Receiver Operating Characteristic Curve.

The ROC curve analysis demonstrated that FAACT A/CS-12 scores had predictive value for future exacerbations (AUC = 0.613, 95%CI = 0.571–0.654, cut off value =40.50), frequent exacerbations (AUC = 0.610, 95%CI = 0.560–0.660, cut off value =32.50), and hospitalizations (AUC = 0.635, 95%CI = 0.589–0.681, cut off value =31.50) (*p* < 0.05) (Tables 5 and 6, Figure 3).

**Table 5.** ROC curves of different indicators predicted exacerbation in COPD patients.

Variables	Exacerbations						Frequent exacerbations					
	AUC	95% CI	p	Cut off	Sensitivity	Specificity	AUC	95% CI	p	Cut off	Sensitivity	Specificity
FAACT A/CS-12 scores	0.613	0.571–0.654	**<0.001**	40.50	59.93	56.14	0.610	0.560–0.660	**<0.001**	32.50	31.36	85.84
FEV1 %pred	0.592	0.549-0.634	**<0.001**	51.65	56.79	60.68	0.584	0.534-0.634	**0.001**	51.65	57.99	57.53
Exacerbations in the past year	0.678	0.639-0.716	**<0.001**	1.50	53.31	73.41	0.642	0.594-0.689	**<0.001**	1.50	53.25	68.06

Notes: The cut-off value was determined at the point where the Youden index (sensitivity + specificity − 1) is maximized.

Abbreviations: AUC: area under the curve; COPD: Chronic Obstructive Pulmonary Disease; FEV1%pred: Forced Expiratory Volume in the first second predicted of percentage; 95% CI: 95% confidence interval; ROC: receiver operating characteristic curve.

**Table 6.** ROC curves of different indicators predicted hospitalization and mortality in COPD patients.

Variables	Hospitalizations						Mortality					
	AUC	95% CI	*p*	Cut off	Sensitivity	Specificity	AUC	95% CI	*p*	Cut off	Sensitivity	Specificity
FAACT A/CS-12 scores	0.635	0.589–0.681	**<0.001**	31.50	31.02	87.43	0.617	0.376-0.857	0.289	29.50	42.86	87.08
FEV1 %pred	0.597	0.549-0.645	**<0.001**	51.45	59.36	59.47	0.589	0.436-0.742	0.415	54.40	85.71	50.14
Exacerbations in the past year	0.645	0.599-0.690	**<0.001**	1.50	52.94	68.67	0.618	0.446-0.790	0.284	0.50	85.71	40.83

Notes: The cut-off value was determined at the point where the Youden index (sensitivity + specificity − 1) is maximized.

Abbreviations: AUC: Area Under Curve; COPD: Chronic Obstructive Pulmonary Disease; FEV1%pred: Forced Expiratory Volume in the first second predicted of percentage; 95%CI: 95% Confidence Interval; ROC: Receiver Operating Characteristic Curve.

## Discussion

In this study, the proportion of male patients was as high as 91.8%. This may have been due to smoking being the primary risk factor for the development of COPD, while smoking rates among females remain relatively low. Furthermore, several studies have shown that the proportion of females with COPD is relatively small in China [20–22].

Anorexia or loss of appetite is common in COPD patients, serves as a surrogate marker of general health status, and is closely linked to COPD prognosis [23]. Understanding the clinical characteristics and prognosis of COPD patients with anorexia is therefore essential for delivering more precise treatment and conducting more comprehensive clinical evaluations. The section A/CS-12 of the FAACT questionnaire, endorsed by the European Society for Clinical Nutrition and Metabolism, is a validated diagnostic tool for anorexia. Its key strength lies in enabling both qualitative and quantitative assessment of anorexia symptoms, with scores ranging from 0 to 48. It is also straightforward and feasible for use in clinical practice. Several studies have identified a FACCT A/CS-12 scores of ≤ 30 as a reliable threshold for diagnosing anorexia [12–14]. Furthermore, lower scores indicate more severe anorexia symptoms.

To our knowledge, this is the first study to quantitatively assess the correlation between anorexia symptoms and clinical characteristics in COPD patients using the FAACT A/CS-12 questionnaire. We found that 17.4% of patients with COPD had anorexia. In addition, we found that patients with anorexia had higher CAT scores, mMRC scores, and CCQ scores, number of exacerbations, and hospitalizations in the past year, while lower FEV1, FEV1%pred, and FEV1/FVC. Furthermore, anorexia scores were positively correlated with FEV1, FEV1%pred, and FEV1/FVC, while negatively correlated with CAT scores, mMRC scores, CCQ scores, and the number of exacerbations, and hospitalizations in the past year in COPD patients with anorexia. In addition, we are the first to report that the patients with anorexia have a higher risk of future exacerbations. These findings suggest that improving anorexia symptoms may help preserve pulmonary function and reduce the risk of future exacerbations or hospitalizations. In fact, previous research has shown that severe anorexia nervosa is associated with emphysematous changes in animal models and may play a primary role in the development of emphysema [24]. Grönberg et al. [7] found that the most frequently reported dietary problems were anorexia symptoms, and related to smoking habits and sex, which differs from our results. This discrepancy may be due to methodological differences: their study involved dietary interviews conducted by trained dietitians, while ours used a quantitative anorexia questionnaire. Additionally, our study had a higher proportion of male patients. In addition, patients with anorexia had a lower BMI. This is expected because reduced food intake leads to weight loss, which contributes to a poorer prognosis in COPD. Our previous study confirmed that patients with COPD and a low BMI had worse pulmonary function, higher symptom burden, and greater risks of hospitalization and mortality [25]. Additionally, this is the first study to demonstrate that FAACT A/CS-12 scores can predict future exacerbations, frequent exacerbations, and hospitalizations, as shown by ROC curve analysis. Although we did not observe a statistically significant difference in mortality between patients with and without anorexia, those with anorexia showed a marked trend toward higher mortality, which may have been due to the relatively short follow-up duration.

This study has several limitations. First, we did not include quantitative computed tomography imaging data. Prior research has shown that computed tomography-measured emphysema indices are significantly higher in patients with anorexia than in controls, and these indices correlate with lung diffusion capacity [26]. Additionally, we did not explore whether anorexia in patients with COPD is associated with central nervous system dysfunction—a topic that warrants investigation in future studies. Finally, larger multicenter studies with longer follow-up periods are needed to further clarify the impact of anorexia on COPD prognosis.

## Conclusions

The COPD patients with anorexia had worse pulmonary function, a higher burden of symptoms, and a higher risk of exacerbation and hospitalization. Clinicians should pay more attention to patients with anorexia and consider implementing appropriate interventions.

## Supplementary Material

Supplement tables and figures revised.docx

## Data Availability

All data in this study are available from the corresponding author Ping Chen for reasonable requests (http://120.77.177.175:9007/a/login).
