# Supplementary material for: The clinical characteristics and prognosis in chronic obstructive pulmonary disease patients with anorexia
Source: Ann Med. 2025 Nov 21;57(1):2590186. doi: 10.1080/07853890.2025.2590186 (PMC12642890; doi:10.1080/07853890.2025.2590186)
Supplement: Supplement tables and figures revised.docx [file IANN_A_2590186_SM4180.docx]

**Supplement table 1. Multivariate analysis for prognosis of COPD patients with anorexia.**

| **Variables** | **Exacerbations** | | **Frequent exacerbations** | | **Hospitalizations** | | **Mortality** | |
| --- | --- | --- | --- | --- | --- | --- | --- | --- |
|  | **OR (95% CI)** | **p value** | **OR (95% CI)** | **p value** | **OR (95% CI)** | **p value** | **OR (95% CI)** | **p value** |
| Groups |  |  |  |  |  |  |  |  |
| Non-anorexia | Reference |  | Reference |  | Reference |  | N/A |  |
| Anorexia | 1.79 (1.12-2.84) | **0.014** | 2.40 (1.48-3.88) | **< 0.001** | 2.25 (1.41-3.59) | **0.001** | N/A | N/A |
| Age | 1.01 (0.99-1.03) | 0.281 | 1.00 (0.97-1.02) | 0.946 | 1.02 (0.99-1.04) | 0.195 | N/A | N/A |
| Sex |  |  |  |  |  |  |  |  |
| Male | Reference |  | Reference |  | Reference |  | N/A | N/A |
| Female | 2.29 (1.05-4.98) | **0.036** | 1.73 (0.75-3.95) | 0.197 | 1.38 (0.60-3.14) | 0.447 | N/A | N/A |
| Education level |  |  |  |  |  |  |  |  |
| Elementary school | Reference |  | Reference |  | Reference |  | N/A |  |
| Junior high school | 1.12 (0.76-1.65) | 0.570 | 1.20 (0.78-1.87) | 0.408 | 0.78 (0.51-1.18) | 0.239 | N/A | N/A |
| High school | 1.41 (0.87-2.29) | 0.160 | 1.37 (0.80-2.34) | 0.247 | 0.91 (0.54-1.54) | 0.732 | N/A | N/A |
| University | 0.69 (0.29-1.61) | 0.388 | 0.51 (0.16-1.58) | 0.243 | 0.69 (0.27-1.77) | 0.440 | N/A | N/A |
| BMI | 0.96 (0.91-1.01) | 0.131 | 0.99 (0.94-1.05) | 0.703 | 1.01 (0.95-1.06) | 0.811 | N/A | N/A |
| Smoke status |  |  |  |  |  |  |  |  |
| Never-smoker | Reference |  | Reference |  | Reference |  | N/A |  |
| Former-smoker | 1.26 (0.68-2.33) | 0.468 | 0.84 (0.43-1.66) | 0.620 | 0.70 (0.36-1.35) | 0.281 | N/A | N/A |
| Current-smoker | 1.21 (0.67-2.20) | 0.530 | 0.91 (0.48-1.75) | 0.787 | 1.01 (0.54-1.91) | 0.964 | N/A | N/A |
| Biofuel exposure |  |  |  |  |  |  |  |  |
| No | Reference |  | Reference |  | Reference |  | N/A |  |
| Yes | 1.03 (0.68-1.57) | 0.884 | 1.17 (0.73-1.86) | 0.521 | 1.07 (0.68-1.69) | 0.764 | N/A | N/A |
| FEV1 %pred | 0.98 (0.97-1.00) | **0.021** | 0.98 (0.96-1.00) | **0.031** | 0.99 (0.98-1.01) | 0.399 | N/A | N/A |
| FEV1/FVC | 1.02 (0.99-1.04) | 0.205 | 1.02 (0.99-1.05) | 0.180 | 0.99 (0.97-1.02) | 0.695 | N/A | N/A |
| CAT | 1.00 (0.96-1.04) | 0.981 | 1.00 (0.95-1.04) | 0.813 | 0.99 (0.95-1.03) | 0.520 | N/A | N/A |
| mMRC | 0.89 (0.71-1.11) | 0.305 | 0.89 (0.69-1.15) | 0.383 | 1.06 (0.83-1.36) | 0.645 | N/A | N/A |
| Exacerbations in the past year | 1.25 (1.16-1.34) | **< 0.001** | 1.17 (1.09-1.25) | **< 0.001** | 1.15 (1.07-1.23) | **< 0.001** | N/A | N/A |
| Therapy |  |  |  |  |  |  |  |  |
| LAMA | Reference |  | Reference |  | Reference |  | N/A |  |
| LABA+ICS | 1.66 (0.62-4.45) | 0.309 | 1.68 (0.47-5.99) | 0.427 | 3.65 (1.11-12.05) | 0.034 | N/A | N/A |
| LABA+LAMA | 1.12 (0.52-2.43) | 0.771 | 2.01 (0.72-5.62) | 0.186 | 1.54 (0.55-4.33) | 0.411 | N/A | N/A |
| LABA+LAMA+ICS | 1.15 (0.53-2.49) | 0.728 | 1.73 (0.61-4.86) | 0.301 | 1.89 (0.67-5.27) | 0.227 | N/A | N/A |
| Others^♥^ | 0.30 (0.06-1.59) | 0.157 | 0.44 (0.05-4.25) | 0.476 | 0.49 (0.05-4.74) | 0.536 | N/A | N/A |
| Prescription outcomes |  |  |  |  |  |  |  |  |
| Adjustment treatment | Reference |  | Reference |  | Reference |  | N/A |  |
| Continuous using | 0.79 (0.52-1.19) | 0.261 | 0.84 (0.53-1.34) | 0.462 | 1.25 (0.77-2.02) | 0.370 | N/A | N/A |

**Notes:** Factors in the logistic model: Anorexia group, education level, therapy, prescription outcome, age, sex, BMI, smoke status, biofuel exposure, FEV1 %pred, FEV1/FVC, CAT, mMRC, and exacerbations in the past year. The bold p values indicate statistical significance. ^♥^ others included SAMA, SABA, SAMA+SABA, LAMA+ICS, ICS and no inhalation therapy.

**Abbreviations:** BMI, Body Mass Index; COPD, Chronic Obstructive Pulmonary Disease; CAT, COPD Assessment Test; FEV1 %pred, Forced Expiratory Volume in the first second predicted of percentage; FVC, Forced Vital Capacity; GOLD, Global Initiative for Chronic Obstructive Lung Disease; ICS, Inhaled Corticosteroids; LAMA, Long-Acting Muscarinic Antagonist; LABA, Long-Acting β2-Agonist; mMRC, modiﬁed Medical Research Council; CCQ, Clinical COPD Questionnaire; A/CS, anorexia/cachexia subscale; FAACT, Functional Assessment of Anorexia/Cachexia Therapy; SABA, Short Acting Beta Agonist; SAMA, Short-Acting Muscarinic Antagonists.

**Supplement table 2. The clinical characteristics of the COPD patients with anorexia after propensity score matching.**

| **Variables** | **Propensity score matching** | | **p value** |
| --- | --- | --- | --- |
|  | **Non-anorexia (n=92)** | **Anorexia (n=96)** |  |
| Age, (years) (Mean ± SD) | 64.8 ± 9.0 | 66.0 ± 7.9 | 0.334 |
| Sex, n (%) |  |  | 0.304 |
| Male | 87 (94.6) | 87 (90.6) |  |
| Female | 5 (5.4) | 9 (9.4) |  |
| Education level, n (%) |  |  | 0.287 |
| Elementary school | 38 (41.3) | 47 (49.0) |  |
| Junior high school | 39 (42.4) | 28 (29.2) |  |
| High school | 12 (13.0) | 16 (16.7) |  |
| University | 3 (3.3) | 5 (5.1) |  |
| BMI, (kg/m^2^), (Mean ± SD) | 21.2 ± 3.5 | 21.1 ± 3.0 | 0.889 |
| Smoke status, n (%) |  |  | 0.413 |
| Never-smoker | 16 (17.4) | 14 (14.6) |  |
| Former-smoker | 37 (40.2) | 32 (33.3) |  |
| Current-smoker | 39 (42.4) | 50 (52.1) |  |
| Smoking, (pack/year) (Median, IQR) | 40.0 (25.0, 56.0) | 46.0 (30.0, 60.8) | 0.137 |
| Biofuel exposure, n (%) |  |  | 0.864 |
| Yes | 24 (26.1) | 24 (25.0) |  |
| No | 68 (73.9) | 72 (75.0) |  |
| FEV1 %pred, (Mean ± SD) | 50.7 ± 20.4 | 53.1 ± 21.6 | 0.442 |
| FEV1/FVC, (Mean ± SD) | 47.0 ± 12.3 | 48.5 ± .12.5 | 0.408 |
| GOLD grades, n (%) |  |  | 0.103 |
| 1 | 10 (10.9) | 12 (12.5) |  |
| 2 | 36 (39.1) | 33 (34.4) |  |
| 3 | 29 (31.5) | 43 (44.8) |  |
| 4 | 17 (18.5) | 8 (8.3) |  |
| GOLD groups, n (%) |  |  | 0.780 |
| A | 7 (7.6) | 6 (6.2) |  |
| B | 33 (35.9) | 31 (32.3) |  |
| E | 52 (56.5) | 59 (61.5) |  |
| CAT, (Mean ± SD) | 16.3 ± 5.9 | 16.2 ± 5.6 | 0.930 |
| mMRC, (Median, IQR) | 2.0 (2.0, 2.0) | 2.0 (1.0, 3.0) | 0.581 |
| Therapy, n (%) |  |  | 0.513 |
| LAMA | 4 (4.3) | 5 (5.2) |  |
| LABA+ICS | 2 (2.2) | 6 (6.2) |  |
| LABA+LAMA | 30 (32.6) | 26 (27.1) |  |
| LABA+LAMA+ICS | 55 (59.8) | 59 (61.5) |  |
| ^♥^ Others | 1 (1.1) | 0 (0.0) |  |
| Exacerbations in the past year, (Median, IQR) | 1.0 (0.0, 3.2) | 1.0 (0.0, 4.0) | 0.394 |
| Hospitalizations in the past year, (Median, IQR) | 0.0 (0.0, 1.0) | 0.0 (0.0, 1.2) | 0.647 |

**Notes:** ^♥^ Others included SAMA, SABA, SAMA+SABA, LAMA+ICS, ICS and no inhalation therapy.

**Abbreviations:** BMI, Body Mass Index; COPD, Chronic Obstructive Pulmonary Disease; CAT, COPD Assessment Test; FEV1, Forced Expiratory Volume in one second; FEV1 %pred, Forced Expiratory Volume in the first second predicted of percentage; FVC, Forced Vital Capacity; GOLD, Global Initiative for Chronic Obstructive Lung Disease; ICS, Inhaled Corticosteroids; IQR, Interquartile Range; LAMA, Long-Acting Muscarinic Antagonist; LABA, Long-Acting β2-Agonist; mMRC, modiﬁed Medical Research Council; SD, Standard Deviation; CCQ, Clinical COPD Questionnaire; A/CS, anorexia/cachexia subscale; FAACT, Functional Assessment of Anorexia/Cachexia Therapy; SABA, Short Acting Beta Agonist; SAMA, Short-Acting Muscarinic Antagonists.

**Supplement table 3. The future exacerbation and mortality of COPD patients with anorexia during one year of follow-up after propensity score matching.**

| **Variables** | **Total**  **(N=188)** | **Propensity score matching** | | **p**  **value** |
| --- | --- | --- | --- | --- |
|  |  | **Non-anorexia (n=92)** | **Anorexia (n=96)** |  |
| Exacerbations during one year, (Median, IQR) | 0.0 (0.0, 2.0) | 0.0 (0.0, 1.0) | 1.0 (0.0, 2.0) | **0.005** |
| Exacerbations, n (%) |  |  |  | **0.014** |
| No | 97 (53.0) | 56 (62.2) | 41 (44.1) |  |
| Yes | 86 (47.0) | 34 (37.8) | 52 (55.9) |  |
| Frequent exacerbations, n (%) |  |  |  | **0.014** |
| No | 129 (70.5) | 71 (78.9) | 58 (62.4) |  |
| Yes | 54 (29.5) | 19 (21.1) | 35 (37.6) |  |
| Hospitalizations during one year, (Median, IQR) | 0.0 (0.0, 1.0) | 0.0 (0.0, 0.0) | 0.0 (0.0, 1.0) | **<0.001** |
| Hospitalizations, n (%) |  |  |  | **<0.001** |
| No | 122 (66.7) | 71 (78.9) | 51 (54.8) |  |
| Yes | 61 (33.3) | 19 (21.1) | 42 (45.2) |  |
| Mortality, n (%) | 7 (1.0) | 2 (2.2) | 3 (3.1) | 1.000 |
| prescription outcome, n (%) |  |  |  | 0.278 |
| Adjustment treatment | 138 (19.1) | 13 (14.0) | 18 (20.0) |  |
| Continuous using | 583 (80.9) | 80 (86.0) | 72 (80.0) |  |

**Notes:** The bold p value indicates statistical significance.

**Abbreviations:** COPD, Chronic Obstructive Pulmonary Disease; IQR, Interquartile Range.


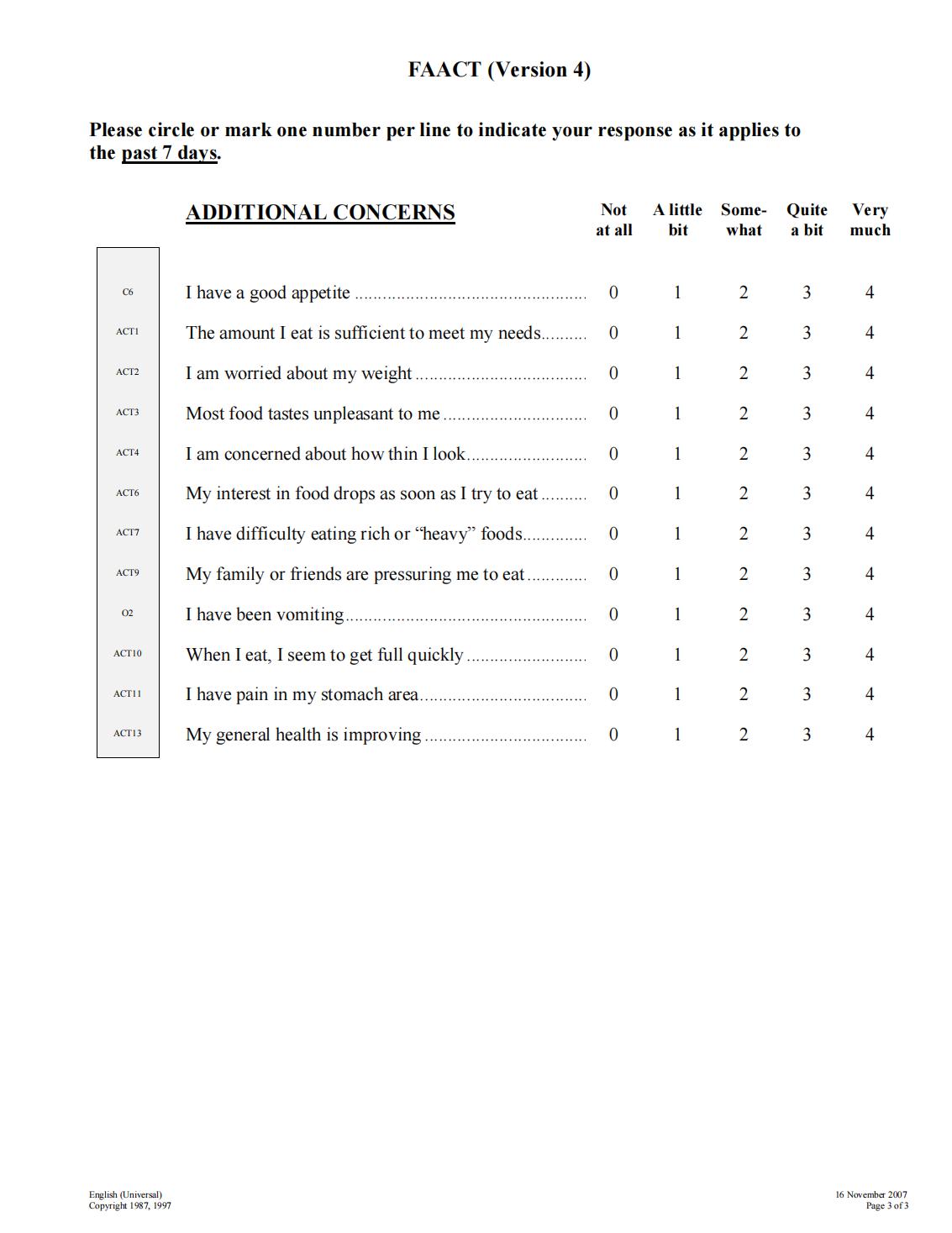


**Supplement figure 1.** The FAACT A/CS-12 questionnaire.


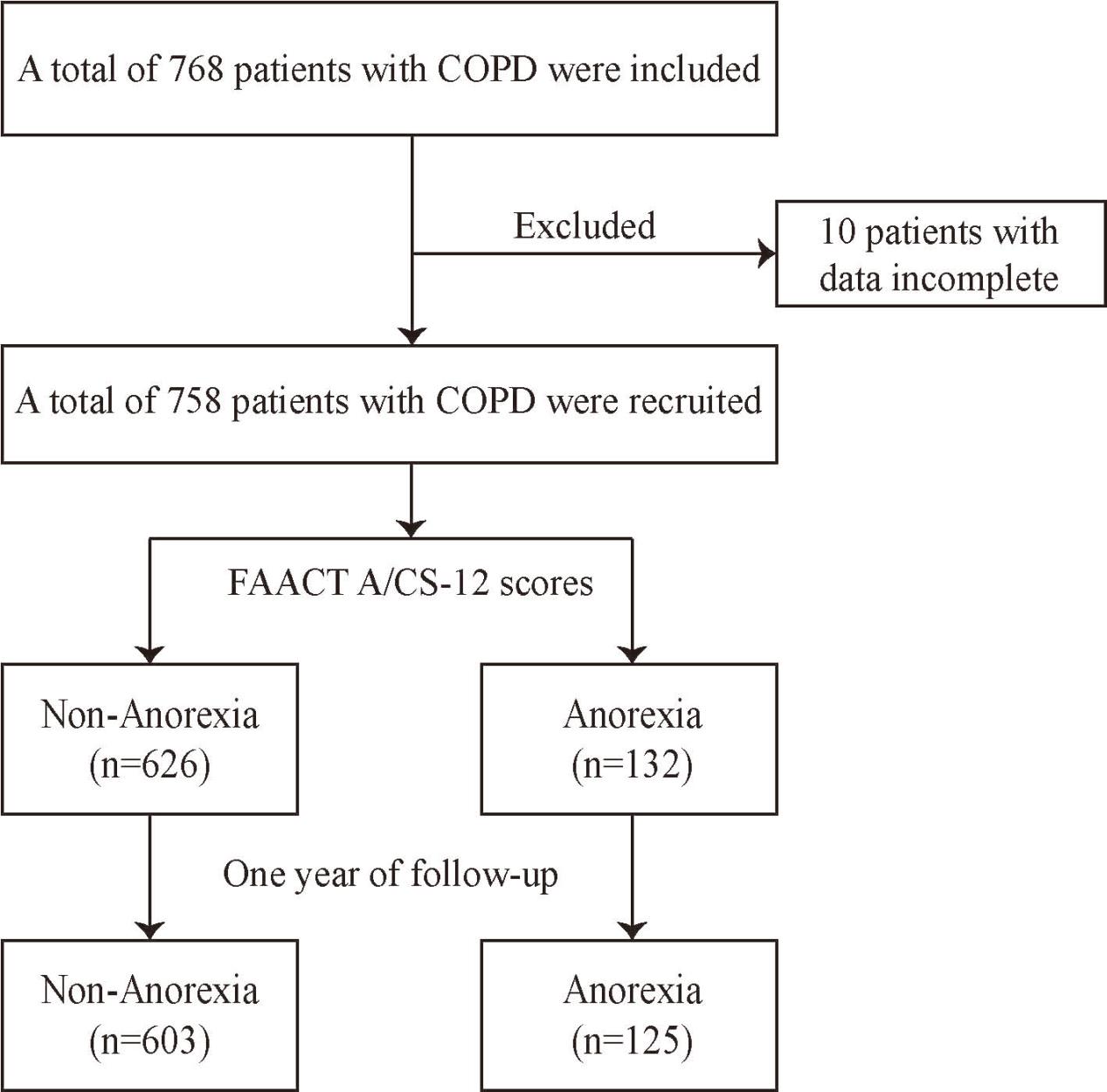


**Supplement figure 2.** Flow chart of this study. A/CS, Anorexia/Cachexia Subscale; COPD, Chronic Obstructive Pulmonary Disease; FAACT, Functional Assessment of Anorexia/Cachexia Therapy.
